# Adolescent adaptive behavior profiles in Williams–Beuren syndrome, Down syndrome, and autism spectrum disorder

**DOI:** 10.1186/s13034-017-0177-0

**Published:** 2017-07-24

**Authors:** Carolina Grego Del Cole, Sheila Cavalcante Caetano, Wagner Ribeiro, Arthur Melo E. e. Kümmer, Andrea Parolin Jackowski

**Affiliations:** 1 Laboratório Interdisciplinar de Neurociências Clínicas (LiNC), Departamento de Psiquiatria, Universidade Federal de São Paulo, Edifício de Pesquisas II – UNIFESP, Rua Pedro de Toledo, 669-3° andar fundos, Vila Clementino, São Paulo, SP Brazil; 2 Unidade de Psiquiatria da Infância e Adolescência (UPIA), Departamento de Psiquiatria, Universidade Federal de São Paulo, Rua Borges Lagoa, 570-8° andar Vila Clementino, São Paulo, SP Brazil; 3 London School of Economics and Political Science, Houghton Street, WC2A2AE London, UK; 4 Departamento de Saúde Mental da, Universidade Federal de Minas Gerais, Av. Prof. Alfredo Balena, 190-sala 235, Belo Horizonte, MG Brazil

## Abstract

**Background:**

Adaptive behavior can be impaired in different neurodevelopmental disorders and may be influenced by confounding factors, such as intelligence quotient (IQ) and socioeconomic classification. Our main objective was to verify whether adaptive behavior profiles differ in three conditions—Williams Beuren syndrome (WBS), Down syndrome (DS), and autism spectrum disorder (ASD), as compared with healthy controls (HC) and with each other. Although the literature points towards each disorder having a characteristic profile, no study has compared profiles to establish the specificity of each one. A secondary objective was to explore potential interactions between the conditions and socioeconomic status, and whether this had any effect on adaptive behavior profiles.

**Methods:**

One hundred and five adolescents were included in the study. All adolescents underwent the following evaluations: the Vineland Adaptive Behavior Scale (VABS), the Wechsler Intelligence Scale for Children (WISC), and the Brazilian Economic Classification Criteria.

**Results:**

Our results demonstrated that the WBS group performed better than the DS group in the communication domain, β = −15.08, t(3.45), p = .005, and better than the ASD group in the socialization domain, β = 8.92, t(−2.08), p = .013. The DS group also performed better than the ASD group in socialization, β = 16.98, t(−2.32), p = .024. IQ was an important confounding factor, and socioeconomic status had an important effect on the adaptive behavior of all groups.

**Conclusions:**

There is a heterogeneity regarding adaptive behavior profiles in WBS, DS, and ASD. These data are important to better design specific strategies related to the health and social care of each particular group.

## Background

This study proposed to analyze differences in the performance of adaptive behavior between groups with genetic syndromes and neuropsychiatric disorders, such as is the case for Williams–Beuren syndrome (WBS), Down syndrome (DS), and autism spectrum disorder (ASD). These groups were also compared with a health control (HC) group. In addition, we have been concerned with analyzing some of the variables that may interfere with the performance of adaptive behavior, such as IQ and socioeconomic level.

### Adaptive behavior

Adaptive behavior is the collection of conceptual, social, and practical skills that have been learned and are performed by people in order to function in their everyday lives [1]. A number of instruments are used to measure adaptive behavior, some of the more widely used ones include the following: the Vineland Adaptive Behavior Scales (VABS) [2], the Adaptive Behavior Assessment System [3], and the Scales of Independent Behavior—revised [4]. The VABS is composed of three main domains for adolescents: communication, daily living and socialization with each domain having three subdomains. The communication domain comprises receptive, expressive and written subdomains; the daily living skills domain comprises personal, domestic and community subdomains; and the socialization domain comprises interpersonal relationships, play, and leisure time and coping skills subdomains [2]. We chose to use VABS in this study because this instrument has been the most widely used of measures of adaptive functioning and semi-structured parent interview over checklists are less vulnerable to reporter bias [5].

Measuring adaptive behavior is of the utmost importance as it provides useful clinical information for the diagnosis of intellectual disabilities, seeing that, limitations in adaptive behavior, associated with deficits in intellectual functioning and age of onset prior to age 18, define intellectual disability [2, 6]. Furthermore, the adaptive behavior performance provides an information that allow establishing education and rehabilitation goals, once that, allow understanding of human functioning [2, 6]. For this reason, it is very important that there is a connection between policy and neurodevelopmental disorders research [7]. It is well known that individuals with certain genetic syndromes share not only physical features but also cognitive and behavioral characteristics. Indeed, different adaptive behavior profiles have been proposed for some genetic syndromes and neuropsychiatric disorders, such as is the case for WBS, DS, and ASD [8–11].

### Williams–Beuren syndrome

Williams–Beuren syndrome is a multisystem genomic disorder, characterized by dysmorphic facial features, short stature, connective tissue abnormalities, and infantile hypercalcemia. People with WBS also have a specific cognitive and behavioral profile, which commonly includes mild intellectual disability (with a relative strength in language and verbal short term memory, and a weakness in visuospatial skills), hypersociality, attention deficit, and anxiety [12]. The condition is caused by the deletion of approximately 26–28 genes from chromosome 7 (7q11.23), and has a prevalence of 1 in 7500 [13]. This syndrome has complex medical, developmental, and behavioral features, requiring intensive multidisciplinary intervention [14].

Compared to healthy controls (HC), individuals with WBS have impairments in adaptive functioning [10]. Moreover, adaptive behavior has been observed to significantly decrease over time in adolescents and adults with WBS [15]. However, there is evidence of heterogeneity, with some individuals functioning at an extremely low level while others function at a chronological age-appropriate level. This variability is likely to reflect inherent and environmental factors [16]. Regarding adaptive behavior profiles, adolescents and adults with WBS usually demonstrate better socialization [17–19] and communication skills but have a weaker performance in the daily living domain [19–21].

### Down syndrome

Down syndrome is the most common autosomal abnormality in humans, with an incidence of 1 in every 800–1200 live births [22]. DS is not only characterized by classic phenotypic physical features, but also by its behavioral and cognitive profile including: intellectual impairment, other cognitive deficits (primarily in speech, language production, and auditory short-term memory) and difficulties in adaptive function [23]. Individuals with DS between 1.08 and 11.5 years age may present better adaptive behavior performance in social skills than in the daily living and communication domains [24]. In addition, significant effects of IQ level were observed on adaptive behavior in most functional skill areas such as communication, community use, functional academics, home living, health and safety, self-direction, social skills, and overall adaptive behavior score in young adults with DS. Participants with a higher IQ showed better outcomes in adaptive behavior and thus better competence in daily living [25].

### Autism spectrum disorder

Autism spectrum disorder is an early-onset neurodevelopmental disorder whose prevalence is estimated to be 11.3 per 1000 [26]. Communication and socialization deficits are common features of individuals with ASD, who tend to respond inappropriately in conversations, to misread nonverbal interactions, and to exhibit impaired ability in building age-appropriate friendships, as well as usually being. Overly dependent on routine, highly sensitive to changes in their environment, or intensely focused on inappropriate items [27]. Intellectual disability may be one of the comorbidities in ASD [28]. Thus, measuring intellectual functioning allows differentiation between high- and low-functioning individuals. Furthermore, adaptive functioning positively correlates with intellectual profile, especially in the communication domain in ASD [29]. People with ASD between ages 4–23 years tend to demonstrate a better performance in communication and daily living, but a weaker performance in the socialization domain [30, 31]. A study comparing 40 high-functioning individuals with ASD with 30 healthy controls, both between 12 and 21 years of age, revealed global adaptive behavior deficits in ASD, with particularly prominent social skills impairments [32].

As the current emphasis of healthcare is on functional outcome, more information is needed regarding the various factors contributing to an individual’s real life functioning and adaptive behavior [33]. Although the literature points towards a specific adaptive behavior profile for each disorder, there is no study comparing the adaptive behavior profiles among WBS, DS and ASD in order to verify the specificity of each profile. Several studies have shown that cognitive functions predict adaptive behavior performance [34–36]. The main objective of the present study was therefore to verify whether adaptive behavior profiles differ across diagnostic groups (WBS, DS, ASD), as compared with healthy controls and with each other.

A secondary objective was to explore the potential relationship between the conditions and the individuals’ socioeconomic status, and the effect on adaptive behavior profiles. Previous studies have demonstrated a strong relationship between socioeconomic status and expressive communication during preschool years through third grade [37, 38]. Parental behavior can also be affected by socioeconomic status, consequently, there is effect on children. LeVine suggested that parental behavior is adapted to socioeconomic and demographic conditions [39]. However, in respect of WBS a study by Hahn et al. [20] found that income or maternal level of education did not influence performance in the communication domain or expressive communication subdomain in WBS or in a developmental disabilities group, with the exception of a statistically significant association between expressive communication and maternal level of education in the developmental disabilities group [20].

## Methods

### Participants

One hundred and five adolescents aged 11–16 years old and resident in the State of Sao Paulo, Brazil, were recruited. The sample comprised four groups: (1) 22 adolescents with WBS, (2) 22 adolescents with DS, (3) 37 adolescents with ASD, and (4) 24 healthy controls (HC).

Adolescents with WBS were recruited from the Brazilian Association of Williams–Beuren Syndrome and all individuals presented diagnostic confirmation by cytogenetic analysis by Fluorescence in situ Hybridization (FISH). Of the 28 individuals with WBS registered with the Brazilian Association of Williams–Beuren Syndrome (aged 11–16 years), 24 individuals agreed to participate in this study. One participant with WBS was excluded due to unfinished questionnaires, and socioeconomic class data were missing for another individual.

Individuals with DS were recruited from the Association of Parents and Friends of Exceptional Children, a non-profit organization that provides social services to people with intellectual disability. The diagnosis of DS was confirmed by examining the karyotype in all individuals. One case was excluded due to comorbidity with ASD and another due to missing data.

Adolescents with ASD were recruited from the specialized clinic in ASD of the Child and Adolescent Psychiatric Unit at the Department of Psychiatry of the Federal University of Sao Paulo (UNIFESP) and from two psychosocial care centers for children and adolescents. Psychosocial care centers for children and adolescents are the main centers for the diagnosis and treatment of children and adolescents with ASD in Brazil. Specialized and experienced professionals in the field carried out the diagnosis of ASD in accordance with the DSM-IV diagnostic criteria, as these assessments took place between 2011 and 2013. One participant with ASD was excluded because of missing data.

The HC group comprised brothers, cousins and friends of the participants with WBS and this group were recruited from events organized by the Brazilian Association of Williams–Beuren Syndrome. The parents or legal guardians of all participants signed informed consent forms, as did the adolescents. The study was approved by UNIFESP’s Research Ethics Committee.

### Instruments

We used the Vineland Adaptive Behavior Scales (VABS) to measure adaptive behavior. VABS evaluates the ability of individuals to cope with environmental changes, learn everyday skills, and demonstrate independence [2]. The scale is based on a structured interview, in which adaptive behavior information is obtained from a significant caregiver. Completion time was approximately 25–90 min. VABS is organized in a structure with three main domains: communication, daily living, and socialization. The raw scores obtained from the domains were weighted to adjust for chronological age, according to the VABS manual, and standardized to obtain a common metric [2]. VABS results can determine strengths and weaknesses in specific adaptive behavior areas, and the scale has extensive representative normative data as well as strong psychometric properties [2].

To estimate IQ we used the Wechsler Intelligence Scale for Children–Third Edition (WISC-III). This instrument evaluates children between 6 and 16 years of age [40]. We estimated IQ by using the weighted sum of the subtests Cubes and Vocabulary [41].

The Critério de Classificação Econômica Brasil (*Brazil Economic Classification Criteria)* developed by the Associação Brasileira de Empresas de Pesquisa [ABEP] (2011) [42] was used to measure socioeconomic status. The classification estimates the income of Brazilian families living in urban areas by evaluating their consumption of durable goods and also assesses their educational level. The socioeconomic classes range from A (highest income) to E (lowest income) [42].

### Statistical analysis

To describe the sample’s characteristics, the frequency of participants’ responses for each categorical variable was calculated. Continuous variables were described based on measures of central tendency (mean) and dispersion (standard deviation).

Mann–Whitney Wilcoxon post hoc test was used to adjust *p* values when bivariate comparisons of continuous variables were performed, as these variables had a non-normal distribution.

When a comparison between two diagnostic groups resulted in a statistically significant difference in any of the clinical scales, we ran a multivariate linear logistic regression model to control for the effect of demographic characteristics and IQ as potential confounders.

These comparisons resulted in three multivariate linear logistic regression models, in which the following pairs of diagnostic groups were compared: (1) WBS vs. DS, (2) WBS vs. ASD, and (3) DS vs. ADS. We repeated these three models for each of the following outcomes: (1) VABS total score, (2) VABS communication domain, (3) VABS socialization domain, and (4) VABS daily living domain.

Finally, we tested for interactions between diagnoses and socioeconomic classes through linear logistic regression models, controlling for IQ. The Vineland dimension (communication, socialization, and daily living) was defined as a dependent variable, whereas diagnosis, in interaction with economic classes, was entered as an independent variable.

In the linear logistic regression models, HC from the A/B classes were regarded as the reference category. For each diagnosis, when the difference between the A/B and C/D classes (e.g., WBS A/B classes vs. WBS C/B classes) was higher than 20%, we considered that this indicated an interaction between diagnosis and economic class, meaning that differences in performance between A/B and C/D classes were clinically significant. We arbitrarily established 20% as a parameter to define the interaction between diagnosis and economic classes, as this can be considered a clinically significant difference in adaptive behavior performance. Among many statisticians and epidemiologists, it is acceptable to set an “arbitrary” parameter such as this when the literature does not provide established parameters [43, 44].

The level of significance was set at *p* < .05. Effect sizes (Cohen’s *d*) were calculated as the difference between means divided by a pooled standard deviation (difference between HC and WBS, DS, and ASD groups). An effect size of ≤.2 indicates a small change, between .2 and .5 a moderate change, and an effect size ≥.8 a large clinical change [45].

## Results

### Sample characteristics

There were no significant differences in age or socioeconomic class between diagnostic groups (WBS, DS, and ASD) and HC (*p* > .05). However, there was a significant difference in IQ between the HC and the diagnostic groups. For additional details see Table 1. The only significant gender difference was between the ASD and HC groups, χ^2^ (1, *N* = 62) = 21.64, *p* < .001.

**Table 1:** Sociodemographic characteristics of adolescents with WBS, DS, ASD compared to the HC group

Groups	Gender	Economic class		Age	WISC (IQ)
	Male	A/B	C/D	Mean (SD)	Mean (SD)
	*n* (%)	*n* (%)	*n* (%)		
HC	10 (42)	13 (54)	11 (45)	13.54 (1.84)	105.62 (16.77)
WBS	14 (61)	9 (41)	13 (59)	13.60 (1.90)	59.39 (9.07)**
DS	10 (45)	11 (50)	11 (50)	13.31 (1.49)	51.22 (3.62)**
ASD	36 (95)**	24 (63)	14 (36)	13.50 (1.62)	84.42 (28.98)*

*HC* healthy controls, *WBS* Williams–Beuren syndrome, *DS* Down syndrome, *ASD* autism spectrum disorder, *WISC* Wechsler Intelligence Scale for Children, *IQ* intelligence quotient

* *p* < .05; ** *p* < .01

In a second analysis, we compared the WBS, DS, and ASD groups to each other. ASD had a significantly higher proportion of males compared to WBS, χ^2^ (1, *N* = 62) = 11.12, *p* < .001. The proportion of males in the ASD group was also significantly higher than in the DS group, χ^2^ (1, *N* = 62) = 18.92, *p* < .001. The WBS group had a significantly higher IQ than the DS group [*z*(43) = 3.10, *p* = .002]. ASD group average IQ was also significantly higher than that of the DS, *t*(58) = −4.35, *p* < .001, and WBS group *t*(59) = −2.90, *p* = .004.

### Differences in adaptive behavior domains between groups

All three diagnostic groups (WS, DS, and ASD) presented significantly lower scores in all VABS domains and total scores as compared to the HC group. Furthermore, the effect sizes were classified as moderate or large regarding adaptive behavior when we compared the HC group with each diagnostic group (WBS, DS, and ASD) (Table 2).

**Table 2:** Effect size of adaptive behavior means for HC, WBS, DS, and ASD groups

	1	2	3	4	HC vs. WBS	HC vs. DS	HC vs. ASD
	HC	WBS	DS	ASD	Cohen’s d/effect size r	Cohen’s d/effect size r	Cohen’s d/effect size r
	Mean (SD)	Mean (SD)	Mean (SD)	Mean (SD)	(*p*)	(*p*)	(*p*)
Socialization	112.45 (17.40)	60.30 (26.09)	55.86 (17.63)	56.52 (29.28)	2.35/.76^a^ (*p* < .001)	3.22/.85^a^ (*p* < .001)	2.32/.75^a^ (*p* < .001)
Communication	103.66 (13.57)	40.82 (18.95)	25.50 (7.46)	58.97 (28.51)	3.82/.88^b^ (*p* < .001)	7.17/.96^b^ (*p* < .001)	2.00/.70^a^ (*p* < .001)
Daily living	103.33 (18.89)	33.91 (15.57)	27.95 (11.79)	53.13 (30.74)	4.06/.89^b^ (*p* < .001)	4.81/.92^b^ (*p* < .001)	1.97/.70^a^ (*p* < .001)
Total VABS	108.62 (16.42)	41.39 (15.39)	32.54 (8.86)	53.63 (26.30)	3.60/.87^b^ (*p* < .001)	5.76**/**.94^b^ (*p* < .001)	2.50/.78^a^ (*p* < .001)

Socialization, communication, daily living skills of Vineland scale; total of Vineland scale (total VABS)

*HC* healthy controls, *WBS* Williams–Beuren syndrome, *DS* Down syndrome, *ASD* autism spectrum disorder

^a^Medium effect size

^b^Large effect size

Differences between diagnostic groups (WBS, DS, and ASD) in relation to adaptive behavior performance are described in Table 3.

**Table 3:** Comparing adaptive behavior between diagnostic groups (WBS, DS, and ASD)

	Total VABS	Daily living	Socialization	Communication
	Mean (SD)	Mean (SD)	Mean (SD)	Mean (SD)
WBS	41.39 (15.39)*	33.91 (15.57)	60.3 (26.09)	40.82 (18.95)**
DS	32.54 (8.86)	27.95 (11.79)	56.52 (29.28)	25.5 (7.46)
WBS	41.39 (15.39)	33.91 (15.57)*	60.30 (26.09)	40.82 (18.95)*
ASD	53.63 (26.30)	53.13 (30.74)	56.52 (29.28)	58.97 (28.51)
DS	32.54 (8.86)*	27.95 (11.79)*	55.86 (17.63)	25.50 (7.46)**
ASD	53.63 (26.30)	53.13 (30.74)	56.5 (29.3)	58.97 (28.51)

Socialization, communication, daily living skills of Vineland scale; total of Vineland scale (total VABS)

*WBS* Williams–Beuren syndrome, *DS* Down syndrome, *ASD* autism spectrum disorder

* *p* < .05; ** *p* < .01

In multivariate linear logistic regression models, the WBS group still performed better than the DS group in the communication domain [β = −15.08, *t*(−3.45), *p* = .005] and remained better than the ASD group in the socialization domain [β = 8.92, *t*(−2.08), *p* = .013]. The DS group also performed better than the ASD group in the socialization domain [β = 16.98, *t*(−2.32), *p* = .024]. Differences between groups lost statistical significance after IQ was controlled for, meaning that IQ acted as a confounding factor (Table 4).

**Table 4:** Multivariate logistic regression models comparing adaptive behavior between groups adjusting for demographics and IQ

Groups	Coef. (95% CI)
WBS vs. DS	
Socialization	4.25 (−10.97 to 19.47)
Communication	−10.61 (−20.94 to −.28)*
Daily living	−.37 (−10.22 to 9.44)
Total VABS	−2.98 (−11.32 to 5.35)
Socialization	4.25 (−10.97 to 19.47)
WBS vs. ASD	
Socialization	−8.92 (−15.84 to −1.99)*
Communication	−1.14 (−6.72 to 4.44)
Daily living	1.80 (−4.39 to 8.01)
Total VABS	−1.83 (−6.72 to 3.04)
DS vs. ASD	
Socialization	−16.97 (−31.61 to −2.33)*
Communication	7.48 (−2.97 to 17.94)
Daily living	2.83 (−11.13 to 16.80)
Total VABS	−.19 (−10.70 to 10.30)

Socialization, communication, daily living skills of Vineland scale; total of Vineland scale (total VABS); demographics (gender, age, and economic class); intelligence quotient (IQ)

*Coef.* confidence interval, *WBS* Williams–Beuren syndrome, *DS* Down syndrome, *ASD* autism spectrum disorder

* *p* < .05

### Association between adaptive behavior and conditions in relation to socioeconomic class while controlling for IQ

Adolescents with delayed development (WBS, DS, and ASD) showed significant lower adaptive behavior scores when compared with HC from A/B economic classes in the bivariate analyses. The statistical differences were significant at *p* < .001 for all comparisons between groups. However, when controlling for IQ, these differences remained only in a few dependent variables as shown in Table 5.

**Table 5:** Linear logistic regression testing for interaction between diagnoses and economic classes, controlling for IQ

	Communication	Socialization	Daily living
	Coef. (95% CI)	Coef. (95% CI)	Coef. (95% CI)
HC + A/B	–	–	–
HC + C/D	17.78 (.38 to 35.17)	−1.05 (−19.71 to 17.60)	21.49 (3.26 to 39.73)
WBS + A/B	−17.34 (−38.70 to 4.01)	−7.84 (−30.75 to 15.06)	−16.00 (−38.40 to 6.38)
WBS + C/D	−31.80 (−52.04 to −11.57)*	−14.72 (−36.42 to 6.97)	−20.39 (−41.61 to .81)
DS + A/B	−38.39 (−59.58 to −17.19)*	−16.05 (−38.78 to 6.68)	−30.37 (−52.59 to −8.15)*
DS + C/D	−35.79 (−57.56 to −14.01)*	−21.59 (−44.95 to 1.76)	−23.78 (−46.61 to −.95)
ASD + A/B	−20.15 (−35.35 to −4.95)	−31.74 (−48.04 to −15.44)**	−19.89 (−35.83 to −3.96)
ASD + C/D	−29.04 (−46.89 to −11.20)*	−42.52 (−61.66 to −23.38)**	−24.61 (−43.32 to −5.90)

*HC* healthy controls, *WBS* Williams–Beuren syndrome, *DS* Down syndrome, *ASD* autism spectrum disorder, *A/B* A and B economic classes, *C/D* C and D economic classes

* *p* < .05; ** *p* < .01

When differences between A/B and C/D classes were higher than 20%, we considered that there was an interaction between adaptive behavior and economic classes. The following interactions occurred: (1) adolescents with WBS from the C/D classes had scores that were 83.8% lower than those from the A/B classes in communication; 88.5% in socialization; and 26.9% in daily living skills. (2) adolescents with DS from C/D classes had scores that were 34.4% lower than those from A/B classes in the socialization domain, and 21.8% lower in daily living skills. (3) adolescents with ASD from C/D classes had scores that were 44.3% lower than those from A/B classes in communication, 34.1% in socialization, and 24.2% in daily living skills.

## Discussion

The findings of this study suggest that there are distinct adaptive behavior profiles in adolescents with WBS, DS, and ASD. Individuals with WBS had better adaptive behavior skills than individuals with DS, especially in communication, as well as better socialization skills than individuals with ASD. Furthermore, the DS group performed better than the ASD group in the socialization domain. These results remained significant after adjusting for IQ, since the groups were not matched for this variable when selected.

In spite of the limitations in the socialization and communication domains in participants with WBS when compared to the HC group, they still performed better in socialization compared to individuals with ASD, and in communication when compared to individuals with DS. This outcome is consistent with the literature with respect to adolescents and adults with WBS who showed satisfactory performance in social interaction [17, 18] and communication skills and poor performance in the remaining adaptive behavior domains [21]. One study showed that the performance of adolescents with WBS was better than that of adolescents with DS in all VABS domains [34], which is in agreement with the results presented in this study. It is reasonable to suggest that although individuals with WBS within the age range of this study, 7.67–46.41 years of age have an interest in interpersonal relationships (socialization), they still have difficulty performing this skill to the same level as HC [46].

Thus, we suggest that the adaptive behavior profile for WBS, in terms of strongest to weakest performance in the three domains, would be in the order: socialization, communication, daily living skills. Our results agree with the personality description of individuals with WBS as “hypersocial” [47].

Our results regarding the adaptive behavior profile of the DS group corroborate other studies that showed discrete differences between domains but a better performance in the socialization domain [34, 48]. Moreover, the DS group showed better performance in the socialization domain than the ASD group, but a worse performance in the communication domain when compared to individuals with WBS. Our results therefore agree with the pattern suggested by other studies that found relatively stronger social skills but weaker communication in individuals with DS between ages 1–11.5 years [9, 24]. DS acquire their adaptive skills ceiling scores at about the age of 12 years, furthermore, presented lower performance than HC children in 65% approximately [49]. A review study identified that the main difficulties in the DS cognitive profile are in communication, such as: auditory-verbal processing and short-term memory, expressive language, grammar, and pronunciation [7]. Therefore, the adaptive behavior profile for DS in this study would be, in terms of strongest to weakest domains: socialization, daily living skills, communication.

Previous adaptive behavior findings in children, adolescents and young adults with ASD showed significant deficits in the socialization domain, compared to a relative ability in the daily living skills and communication domains [30, 50, 51]. Thus, some results described in the literature corroborate with the clinical definition of ASD, which is characterized by impairment in social development. Accordingly, in our study the WBS and DS groups showed better performance in the socialization domain than the ASD group, after controlling for IQ. The adaptive behavior profile for ASD in this study would be, in terms of strongest to weakest domains: Daily Living Skills > Communication > Socialization.

Subjects with WBS, DS, and ASD presented lower adaptive behavior performance when compared to the HC group. These difficulties in functional performance in all assessed diagnostic groups in this study are likely associated with limitations derived from the disorder itself. For some adaptive behavior difficulties, IQ might play an important role in adolescents with WBS, DS, and ASD, as previous studies have observed a positive correlation between adaptive behavior and IQ in ASD [31, 52–54], DS [25], and WBS [19]. Therefore, the main differences in terms of performance in adaptive behavior between the groups in this study can only be observed when taking into account IQ. For this reason, we believe it is essential describe the influence of intellectual disability in adaptive behavior performance.

Finally, the result of the analysis identified possible associations between adaptive behavior profiles and socioeconomic class, suggesting that being in the C/D classes might be related to increased adaptive behavior impairment. This result corroborates the theory that parental behavior is adapted by socioeconomic and demographic conditions with a subsequent effect on their children [39], with previous studies demonstrating a strong relationship between socioeconomic status and expressive communication during preschool years through third grade [37, 38]. A study evaluating the risk and protective factors of parental well-being compared parents of children with intellectual disability with control children and found that differences in economic hardship and self-rated health were the strongest predictors for well-being [55]. Thus, Olsson and Hwang [55] confirmed the importance of the relationship between socioeconomic status and the well-being of parents of children with intellectual disability.

As there are no parameters in the literature that define a significant difference in the Vineland scores, a difference of 20% was chosen as being clinically significant based on our clinical experience. The proposed 20% can be a good starting point to demonstrate the relationship between socioeconomic class and the condition, and the effect on adaptive behavior. Nonetheless, it would be worthwhile conducting more specific studies to test this parameter, which, if confirmed, possibly could indicate how interventions that improve families’ socioeconomic conditions (i.e., the income and education level) most likely could help to alleviate the effects of genetic syndromes on adaptive behavior.

When interpreting the results of our study, some limitations should be considered. First, the sample size was defined based on the available number of adolescents with WBS, which is a rare syndrome. Despite this limitation, our statistical models were able to demonstrate differences between the groups that were consistent with our hypotheses and with the literature. Second, only two subscales of the WISC-III were used to estimate IQ. Even though it has been shown that this approach provides scores that are similar to those obtained through the full WISC-III, it does not include other dimensions of intellectual function that may be related with adaptive behavior. The third limitation was not having collected information such as type of school (mainstream or specialist school), other co-morbid or medical conditions and maternal education. The final limitation was the gender differences between the ASD group and the other groups. However, our multivariate analyses showed that gender did not play a significant role as a potential predictor of adaptive behavior in our sample, although females with ASD tend to be somewhat more functional than males in relation to adaptive behavior [56]. Future studies should explore in more depth whether adaptive behavior profiles also differ between male and female individuals with ASD.

## Conclusion

Ours results can help devise intervention strategies that optimize developmental independence, family support, and community participation among adolescents with neurodevelopmental disabilities. Further research is necessary to measure the effectiveness of intervention strategies based on the adaptive behavior profiles for each specific condition.

Furthermore, our results also indicate that socioeconomic class has an important effect on adaptive behavior in adolescents with genetic syndromes and ASD. The possible effects of socioeconomic status; parental schooling; levels of family, health and educational support which affect adaptive behavior warrant further investigation.

## References

[1] Schalock RL, Borthwick-Duffy SA, Bradley VJ, Buntinx WH, Coulter DL, et al. Intellectual disability: definition, classification and systems of supports. 11th ed. Washington, DC: American association on intellectual and developmental disabilities; 2010.

[2] Sparrow SS, Balla DA, Cicchetti DV (1984). Vineland Adaptive Behavior Scales.

[3] Burns MK. Test review of the Adaptive Behavior Assessment System. 2nd ed. In: Spies RA, Plake BS (eds.). The sixteenth mental measurement yearbook. New York: Springer; 2005.

[4] Maccow G. Test review of the Scales of Independent Behavior—revised. In: Plake BS, Impara JC. The fourteenth mental measurements yearbook. New York: Oxford University Press; 2001. http://www.unl.edu/buros. Accessed 2 Aug 2015.

[5] Angold A, Costello E. A review of the issues relevant to the creation of a measure of disability in children based on the World Health Organization’s international classification of functioning and disability (ICIDH-2). Prepared for the World Health Organization; 2000.

[6] Tassé MJ, Schalock RL, Balboni G, Bersani H, Borthwick-Duffy SA, Spreat S, Zhang D (2012). The construct of adaptive behavior: its conceptualization, measurement, and use in the field of intellectual disability. Am J Intellect Dev Disabil.

[7] Dykens EM, Hodapp RM (2001). Research in mental retardation: toward an etiologic approach. J Child Psychol Psychiatry.

[8] Stone WL, Ousley OY, Hepburn SL, Hogan KL, Brown CS (1999). Patterns of adaptive behavior in very young children with autism. Am J Ment Retard.

[9] Dykens EM, Hodapp RM, Evans DW (1994). Profiles and Development of Adaptive Behavior in Children with Down. Am J Ment Retard.

[10] Mervis CB, Klein-Tasman BP (2000). Williams syndrome: cognition, personality, and adaptive behavior. Ment Retard Dev Disabil Res Rev.

[11] Mervis CB, Klein-Tasman BP, Mastin ME (2001). Adaptive behavior of 4-through 8-year-old children with Williams syndrome. Am J Ment Retard.

[12] Morris CA (2010). Introduction: williams Syndrome. Am J Med Genet C Semin Med Genet.

[13] Strømme P, Bjømstad PG, Ramstad K (2002). Prevalence estimation of Williams syndrome. J Child Neurol.

[14] Walton JR, Martens MA, Pober BR. The proceedings of the 15th professional conference on Williams Syndrome. Am J Med Genet A. 2017; 173:1159–71. 10.1002/ajmg.a.38156 28371210

[15] Fisher MH, Lense MD, Dykens EM (2016). Longitudinal trajectories of intellectual and adaptive functioning in adolescents and adults with Williams syndrome. J Intellect Disabil Res.

[16] Brawn G, Porter M (2014). Adaptive functioning in Williams syndrome and its relation to demographic variables and family environment. Res Dev Disabil.

[17] Elison S, Stinton C, Howlin P (2010). Health and social outcomes in adults with Williams syndrome: findings from cross-sectional and longitudinal cohorts. Res Dev Disabil.

[18] Howlin P, Davies M, Udwin O (1998). Cognitive functioning in adults with Williams syndrome. J Child Psychol Psychiatry.

[19] Fisch GS, Carpenter N, Howard-Peebles PN, Holden JJ, Tarleton J (2007). Studies of age-correlated features of cognitive-behavioral development in children and adolescents with genetic disorders. Am J Med Genet A.

[20] Hahn LJ, Fidler DJ, Hepburn SL (2014). Adaptive behavior and problem behavior in young children with Williams syndrome. Am J Intellect Dev Disabil.

[21] Greer MK, Brown FR, Pai GS, Choudry SH, Klein AJ (1997). Cognitive, adaptive, and behavioral characteristics of Williams syndrome. Am J Med Genet B Neuropsychiatr Genet.

[22] Pueschel SM, Thuline HC. Chromosome disorders. In: Matson JL, Mulick JA, editors. Handbook of mental retardation. 2nd ed. Elmsford, NY, US: Pergamon Press; 1991. p. 115–138.

[23] Määttä T, Tervo-Määttä T, Taanila A, Kaski M, Iivanainen M (2006). Mental health, behaviour and intellectual abilities of people with Down syndrome. Downs Syndr Res Pract.

[24] Dykens E, Hodapp R, Evans D (2006). Profiles and development of adaptive behavior in children with Down syndrome. Downs Syndr Res Pract.

[25] de Sola S, de la Torre R, Sánchez-Benavides G, Benejam B, Cuenca-Royo A (2015). A new cognitive evaluation battery for Down syndrome and its relevance for clinical trials. Front Psychol.

[26] Baio J. Prevalence of autism spectrum disorders: autism and developmental disabilities monitoring network, 14 sites, United States, 2008. Morbidity and mortality weekly report. Surveillance summaries. Centers for disease control and prevention; 2012. p. 61. 22456193

[27] American Psychiatric Association (2013). Diagnostic and statistical manual of mental disorders.

[28] Centers for Disease Control and Prevention (2014). Prevalence of autism spectrum disorder among children aged 8 years—autism and developmental monitoring network, 11 sites, United States, 2010. MMWR Surveill Summ.

[29] Mouga S, Café C, Almeida J, Marques C, Duque F (2016). Intellectual Profiles in the Autism Spectrum and Other Neurodevelopmental Disorders. J Autism Dev Disord.

[30] Pugliese CE, Anthony L, Strang JF, Dudley K, Wallace GL, Kenworthy L (2015). Increasing adaptive behavior skill deficits from childhood to adolescence in autism spectrum disorder: role of executive function. J Autism Dev Disord.

[31] Kanne SM, Gerber AJ, Quirmbach LM, Sparrow SS, Cicchetti DV, Saulnier CA (2011). The role of adaptive behavior in autism spectrum disorders: implications for functional outcome. J Autism Dev Disord.

[32] Kenworthy L, Case L, Harms MB, Martin A, Wallace GL (2010). Adaptive behavior ratings correlate with symptomatology and IQ among individuals with high-functioning autism spectrum disorders. J Autism Dev Disord.

[33] Chang CL, Yen CF, Yang P (2013). Adaptive behaviors in high-functioning Taiwanese children with autism spectrum disorders: an investigation of the mediating roles of symptom severity and cognitive ability. J Autism Dev Disord.

[34] Di Nuovo S, Buono S (2011). Behavioral phenotypes of genetic syndromes with intellectual disability: comparison of adaptive profiles. Psychiatry Res.

[35] Fu TJ, Lincoln AJ, Bellugi U, Searcy YM (2015). The association of intelligence, visual-motor functioning, and personality characteristics with adaptive behavior in individuals with Williams syndrome. Am J Intellect Dev Disabil.

[36] Gilotty L, Kenworthy L, Sirian L, Black DO, Wagner AE (2002). Adaptive skills and executive function in autism spectrum disorders. Child Neuropsychol.

[37] Stipek DJ, Ryan RH (1997). Economically disadvantaged preschoolers: ready to learn but further to go. Dev Psychol.

[38] Walker D, Greenwood C, Hart B, Carta J (1994). Prediction of school outcomes based on early language production and socioeconomic factors. Child Dev.

[39] LeVine RA (1988). Human parental care: universal goals, cultural strategies, individual behavior. New Dir Child Adolesc Dev.

[40] Wechsler D (1991). WISC-III: Wechsler intelligence scale for children: manual.

[41] de Mello CB, Argollo N, Shayer B, Abreu N, Godinho K (2011). Versão abreviada do WISC-III: correlação entre QI estimado e QI total em crianças brasileiras. Psicologia: Teoria e Pesquisa..

[42] Associação Brasileira de Empresas de Pesquisa: Critério de Classificação Econômica Brasil. 2011. http://www.abep.org/codigosguias/ABEP_CCEB.pdf. Accessed 02 Feb 2011.

[43] Hanks G, Cherny NI, Christakis NA, Kaasa S (2011). Oxford textbook of palliative medicine.

[44] Norleans MX (2000). Statistical methods for clinical trials.

[45] Portney LG, Watkins MP (2009). Foundations of Clinical Research: applications to Practice. Hardcover, Ed.

[46] Freeman K, Williams TI, Farran E, Brown J (2010). Williams Syndrome: the extent of agreement between parent and self report of psychological difficulties. Eur J Psychiatry..

[47] Dykens EM, Rosner BA (1999). Refining behavioral phenotypes: personality-motivation in WBS and Prader-Willi syndrome. Am J Ment Retard.

[48] Chapman RS, Hesketh LJ (2000). Behavioral phenotype of individuals with Down syndrome. Ment Retard Dev Disabil Res Rev.

[49] van Duijn G, Dijkxhoorn Y, Scholte EM, van Berckelaer-Onnes IA (2010). The development of adaptive skills in young people with Down syndrome. J Intellect Disabil Res.

[50] Volkmar FR, Sparrow SS, Goudreau D, Cicchetti DV, Paul R (1987). Social deficits in autism: an operational approach using the Vineland Adaptive Behavior Scales. J Am Acad Child Adolesc Psychiatry.

[51] Szatmari P, Bryson SE, Boyle MH, Streiner DL, Duku E (2003). Predictors of outcome among high functioning children with autism and Asperger syndrome. J Child Psychol Psychiatry.

[52] Burack JA, Volkmar FR (1992). Development of low-and high-functioning autistic children. J Child Psychol Psychiatry.

[53] Klin A, Saulnier CA, Sparrow SS, Cicchetti DV, Volkmar FR (2007). Social and communication abilities and disabilities in higher functioning individuals with autism spectrum disorders: The Vineland and the ADOS. J Autism Dev Disord.

[54] Su CY, Lin YH, Wu YY, Chen CC (2008). The role of cognition and adaptive behavior in employment of people with mental retardation. Res Dev Disabil.

[55] Olsson MB, Hwang CP (2008). Socioeconomic and psychological variables as risk and protective factors for parental well-being in families of children with intellectual disabilities. J Intellect Disabil Res.

[56] Mandic-Maravic V, Pejovic-Milovancevic M, Mitkovic-Voncina M, Kostic M, Aleksic-Hil O (2015). Sex differences in autism spectrum disorders: does sex moderate the pathway from clinical symptoms to adaptive behavior?. Sci Rep.

